# Hydroxymethylation and tumors: can 5-hydroxymethylation be used as a marker for tumor diagnosis and treatment?

**DOI:** 10.1186/s40246-020-00265-5

**Published:** 2020-05-06

**Authors:** Tianmin Xu, Haoyue Gao

**Affiliations:** The Second HospitaI of Jilin University, Changchun, Jilin China

**Keywords:** 5-Hydroxymethylcytosine, 5-Methylcytosine, DNA demethylation, Cancer epigenetics, Cell-free DNA

## Abstract

5-Methylcytosine (5mC) is considered as a common epigenetic modification that plays an important role in the regulation of gene expression. At the same time, 5-hydroxymethylcytosine (5hmC) has been found as an emerging modification of cytosine bases of recent years. Unlike 5mC, global 5hmC levels vary from tissues that have differential distribution both in mammalian tissues and in the genome. DNA hydroxymethylation is the process that 5mC oxidates into 5hmC with the catalysis of TET (ten-eleven translocation) enzymes. It is an essential option of DNA demethylation, which modulates gene expression by adjusting the DNA methylation level. Various factors can regulate the demethylation of DNA, such as environmental toxins and mental stress. In this review, we summarize the progress in the formation of 5hmC, and obtaining 5hmC in a cell-free DNA sample presents multiple advantages and challenges for the subject. Furthermore, the clinical potential for 5hmC modification in dealing with cancer early diagnosis, prognostic evaluation, and prediction of therapeutic effect is also mentioned.

## Background

**Fig. 1:**
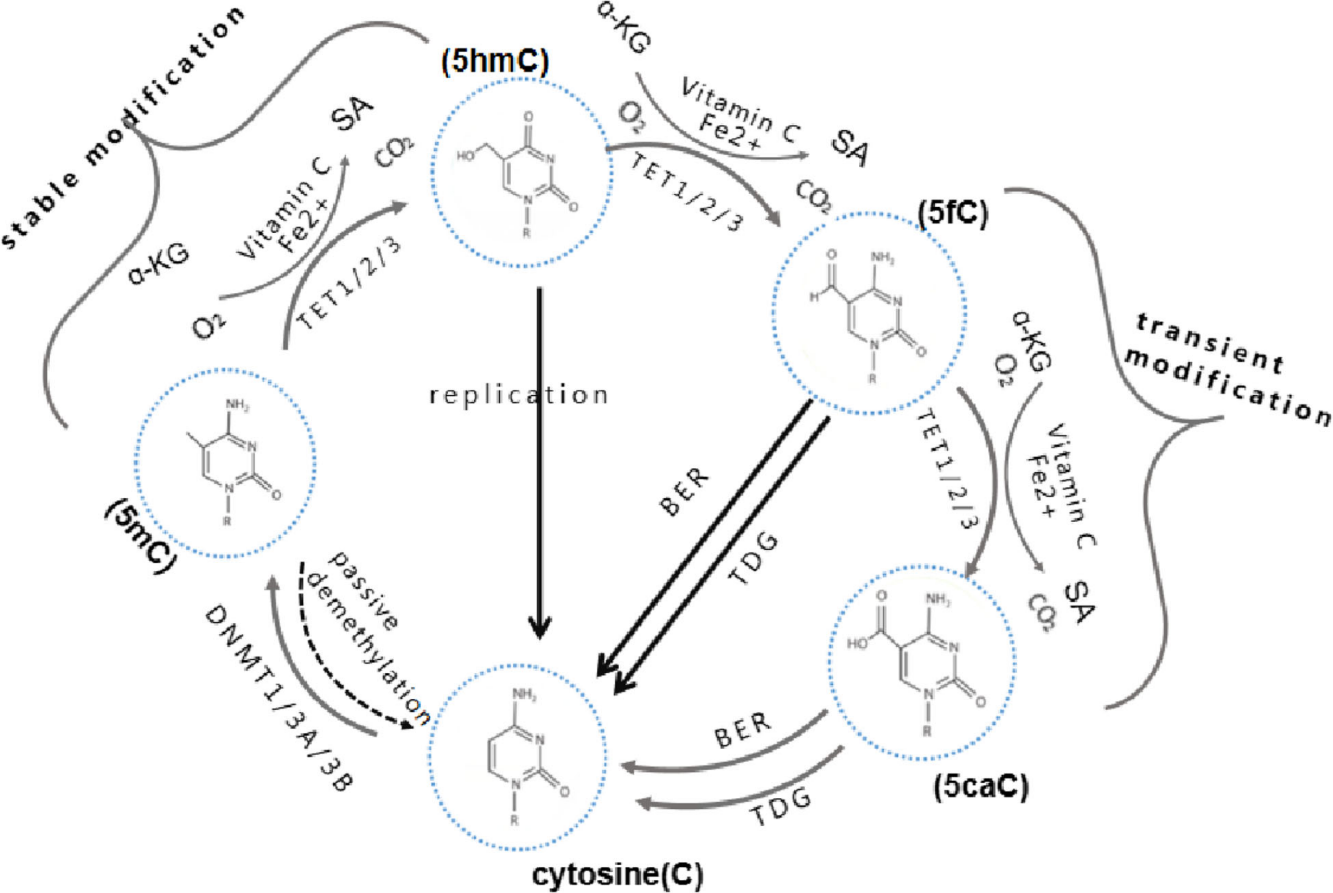
Chemical structures of 5-methylcytosine (5mC) and its oxidized modifications. Cytosine is converted to 5-methylcytosine by both DNMT1 and DNMT3. 5-Methylcytosine is oxidized by TET1, TET2, or TET3 to yield 5-hydroxymethylcytosine (5hmC). By a similar reaction, using the same enzymes, 5-hydroxymethylcytosine is further oxidized to 5-formylcytosine (5fC) and 5-carboxycytosine (5caC). Both 5-formylcytosine and 5-carboxycytosine are depyrimidated by the thymine DNA glycosylase (TDG) and processed by the base excision repair (BER) pathway. All these pathways could result in unmodified cytosine. ɑ-KG = ɑ-ketoglutaric acid; SA = succinic acid

In the process of carcinogenesis, changes in epigenome as early events are generally the same as changes in the genome, which can inactivate tumor suppressor genes and activate proto-oncogenes and play an essential role in the occurrence, development, invasion, and metastasis of tumor [1]. For the past few years, researchers have extended the studies on tumors to the field of epigenetics. During tumor malignancy, distinct epigenetic modifications such as histone modification, DNA methylation, and demethylation, non-coding RNA may become effective biomarkers for high-risk assessment, early diagnosis, prognosis, and reference for drug treatment [2]. In mammals, TET dioxygenase mediates the oxidation of 5mC to 5-hydroxymethylcytosine (5hmC), 5-formylcytosine (5fC), and 5-carboxylcytosine (5caC). Then, replication-dependent 5mC dilution or thymine DNA glycosylase (TDG)-dependent base excision repair (BER) performed. Thus, 5-methylcytosine (5mC) can be reverted to unmodified cytosine (C) [3]. In a series of oxidation reactions, 5-hydroxymethylcytosine (5hmC) is the first product. 5-Methylcytosine (5mC) and α-ketoglutarate (α-KG) formed 5hmC with iron and oxygen as cofactors under the catalysis of ten-eleven translocation (TET) family proteins (Fig. 1). TET enzymes can further oxidize 5hmC to produce 5-formylcytosine and 5-carboxylcytosine [4, 5]. Consistent with their relatively low content in the genome, 5fC and 5caC have poor stability [4–6].

There are two mechanisms of DNA demethylation: active demethylation and passive demethylation [7]. Active demethylation is that TET protein oxidized 5-mC to generate 5-hmC, 5-fC, and 5-caC. 5-fC and 5-caC can directly start base excision repair (BER) to generate cytosine under thymine glycosylase (TDG). Passive demethylation is also called the deamination pathway. In the reaction, 5-mC is converted into thymine under the deaminase, then initiate base excision repair with DNA thymine glycosylase and finally generate cytosine. The main methods of passive demethylation are the following [8]: ① Oxidize the methyl group to carboxyl group and complete demethylation by decarboxylation. ② Remove 5-mC by BER pathway and replace with unmodified cytosine. ③ Specific deaminase recognizes and converts 5-mC to C. The 5-mC was converted to C through deamination to form a mismatch. Then, BER was started under TDG. ④ Demethylation through hydrolysis. 5-hmC plays an essential role in both active demethylation and passive demethylation and is closely related to gene expression regulation. Different reaction processes regulate dynamic DNA demethylation. The availability and content of substrates and cofactors, post-transcription, and post-translation modifications of TET proteins are all factors. Previous studies that focused on structure and biochemical functions have elucidated how TET proteins and TDG mediate active DNA demethylation [8, 9]. Among them, TET proteins may play a different role than the traditional catalytic enzyme. Further analysis is needed to clarify the function of TET proteins themselves and their role in DNA demethylation. Besides, with the continuous development of mapping and tracking technology of 5mC oxidation products, the functions of oxidation products 5-hydroxymethylcytosine (5hmC), 5-formylcytosine (5fC), and 5-carboxylcytosine (5caC) can be studied in depth. Different from 5mC, the content of 5hmC in different tissue has better stability and stronger robustness, which presents tissue specificity, especially showing the differences between cancerous tissue and healthy tissue [10]. Since 5hmC is enriched in promoters, enhancers, and other transcriptional regulatory elements, it is more closely related to gene expression [11, 12]. This leads us to believe that it may have the potential of applying in the clinical diagnosis of tumors, like some vital regulatory molecules of epigenetics, and could be used to predict the metastasis, recurrence, and prognosis. Several surveys indicate that the potency and clinical convenience of tumor biomarkers are essential for the successful delivery of certain drugs and better clinical care for cancer patients. Due to the long incubation period of cancer and symptoms only start to emerge at an advanced stage, effective and timely treatment may not be available. Hence, efficient screening and early detection are particularly crucial to improve patient survival and quality of life [11, 13].

With the rapid development of liquid biopsy technique, cell-free DNA (cfDNA) originating from different tissues and entering blood circulation has become available research in recent years in the field of clinical diagnosis [14]. It only requires blood samples from cancer patients, with advantages of high efficiency, low cost, and smooth operation. From the perspective of patients, compliance is better and dynamic monitoring can be achieved. Most importantly, it makes up for the lack of inadequate acquisition due to the low content of 5hmC in tumor tissues [15, 16]. In this review, we list the diagnostic function of 5hmC in cfDNA in early detection as well as its advantages in anticancer drug selection and efficacy monitoring. Meanwhile, in a variety of different types of cancer, the specific mechanisms by which epigenetic pattern changes identify oncogene mutations and dysregulations remain to be elucidated, and the problem of standardization requirements for all kinds of samples is yet to be solved.

## Advantages and disadvantages of ctDNA as 5hmC research sample

Liquid biopsy is a diagnostic analysis of body fluid samples, such as circulating tumor cells (CTC), circulating tumor DNA (ctDNA), and exosome in the blood [17]. In recent years, ctDNA and cfDNA detection have been drawing much attention in diagnosis and treatment and become the research hotspot, as they have advantages of convenience, minimally invasive, convenient real-time monitoring, and easier to reflect the information of genome-wide tumorigenesis [17]. The methods used in ctDNA and cfDNA detection are known as polymerase chain reaction (PCR) technology (such as ARMS-PCR (amplification refractory mutation system) and digital PCR) and the next-generation sequencing technology (NGS) [18]. Although some ctDNA tests have been proved to have clinical validity and feasibility in analyzing some advanced cancer species, such as colorectal cancer, most of the ctDNA tests lack sufficient evidence to support their clinical practicability [19, 20]. At this point, useful clinical evidence of ctDNA tests in early tumor detection, treatment monitoring, or residual tumor surveillance is limited, and there is also a lack of clinical practicability evidence.

**Fig. 2:**
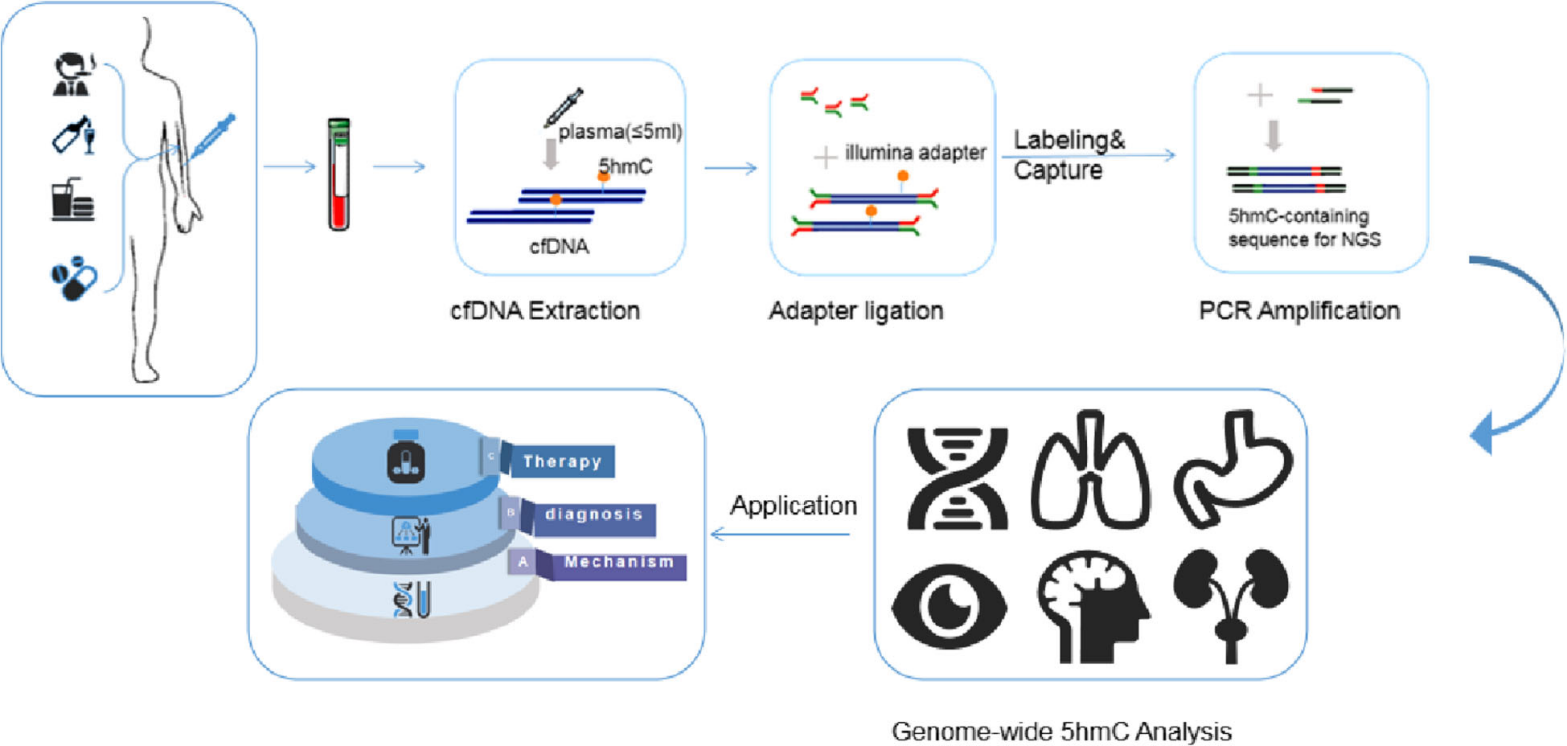
Detecting 5hmC biomarkers in cfDNA of human cancers. The workflow of 5hmC-Seal profiling from cfDNA is shown. Purified cfDNA is ligated with standard sequencing adapters. 5hmC-containing cfDNA fragments are selectively labeled and captured, followed by PCR amplification and next-generation sequencing (NGS)

Given the rapid progress of ctDNA research and clinical tools and guidelines, it will be necessary to re-evaluate the pertinent literature so that standardized guidelines for various indexes of body fluid samples will be available soon [21]. There are many uncertain factors in the pre-processing steps of ctDNA test samples, and these variables may affect the sample purity, so as the subsequent test evaluation [22]. On the whole, many factors may affect the final result in the following steps, including blood drawing, collecting, processing, storage, transportation, and DNA extraction and purification. Also, biological factors related to patients may affect the release of cell-free DNA through the bloodstream, such as smoking, anemia, non-malignant diseases like inflammation, and autoimmune diseases (Fig. 2). However, the technology of sample control is still limited and many problems remain to be solved [21, 23]. To determine whether ctDNA can be used as an active marker in clinical practice, it is necessary to comprehensively evaluate its accuracy, sensitivity, specificity, and repeatability [24, 25]. At present, the method for assessing the accuracy and uniqueness of the plasma cfDNA test is mainly through methodological comparison with paired tissue samples [26]. Studies have shown that there may be differences between plasma ctDNA tests and biopsy results, which may be caused either by the performance of analysis (such as the analysis sensitivity) or by other biological properties. Multiple factors such as cancer type, tumor stage, temporal and spatial heterogeneity of tumor, and different sampling time between tissue and plasma can cause differences. Due to the low content of ctDNA in plasma, it is significant to explore the lower detection limit [27]. Considering the impact of biological property and nonbiological factors above, it is more objective and recommended to verify the detectability near the detection limit by incorporating cell lines or artificially constructing standardized samples [28]. The minimum analytical sensitivity and specificity required for ctDNA tests should be established on specific intended uses to facilitate its clinical application while emphasizing that mutation results must be combined with other clinical information to adjust the therapeutic regimen. The proportion of ctDNA in plasma cell-free DNA (also known as purity) varies from person to person. Similarly, the percentage of ctDNA in total cell-free DNA in different patients also has significant differences [29, 30]. This explains why we need to study further the prognostic significance of mutation abundance in ctDNA tests.

As epigenetic changes in cell-free DNA (cfDNA) have been widely observed to show high sensitivity and specificity in disease detection and classification, including 5-methylcytosine (5mC) and 5-methylcytosine (5hmC), it is vital to collect and integrate the bioinformation of cfDNA for future clinical application. However, due to the lack of effective collection, standardized quality control, and analysis procedures, the clinical utilization of these data cannot be effectively realized. CFEA (http://www.bio-data.cn/ CFEA) is a cell-free epigenome database used to involve 5mC, 5hmC, and NP (nucleosomal localization) of 27 kinds of human diseases. Bioinformation was quality controlled and standardized in the database. Besides, relevant clinical information was collected to facilitate the search and download of cell-free epigenome data. Users can better view and compare the development of the cfDNA epigenome in different stages of the tumor. The comprehensive and timely CFEA database has excellent potential for the development of liquid biopsy biomarkers for various human diseases [16].

## The value of 5hmC in tumor diagnosis

Produced under the active demethylation mediated by the TET (ten-eleven transposition) dioxygenase family, 5mC is modified and oxidized to 5-hydroxymethyl cytosine (5hmC) and further oxidized to 5-formylcytosine (5fC) and 5-carboxycytosine (5caC) [10]. As a relatively stable DNA marker, 5hmC plays a role as a gene expression marker in the whole genome sequencing map of mammalian cells and tissues [31]. Since 5hmC is mainly distributed in the active transcriptional region, accompanied by open chromatin, and allowed histone modification, it is considered to be closely related to gene expression, which can be an ideal candidate biomarker with high chemical stability for cancer diagnosis [14, 32]. When 5mC in cfDNA was sequenced with bisulfite as a high background level inhibitory marker, the results were severely disturbed by DNA degradation [33]. However, unlike 5mC, 5hmC can be mapped with a low level of DNA and highly sensitive selective chemical markers, and the identified 5hmC has cancer type specificity [34–36]. Changes in 5hmC rich in enhancers, gene bodies, and promoters are closely related to changes in gene expression levels [37]. They can reproduce changes in gene expression in different cell states. Therefore, it can be combined with 5mC in cfDNA of liquid biopsy for non-invasive diagnosis and prognosis. Sensitive detection of cytosine modification patterns is helpful to identify cancer-specific biomarkers, and dynamic monitoring 5hmC needs to be put into clinical use because of the lower cost of obtaining blood samples, higher patient compliance, and clinical convenience [38].

Further, researchers found that in lung cancer patients, the content of 5hmC in cfDNA decreased gradually, while in the liver and pancreatic cancer patients, cell-free hydroxymethylome had their disease-specific changes [14, 26, 39]. However, for HCC (hepatocellular carcinoma), after calculating the HCC scores of postoperative and recurrent HCC samples, the researchers learned that HCC scores could accurately follow up on the treatment and understand the recurrence status of patients [40, 41]. In combination with the HCC score, cell-free 5hmC sequencing provides an opportunity to detect HCC, monitor treatment outcomes, and monitor recurrence status [42]. The unbiased gene-level analysis of tSNE (t-distributed stochastic neighbor embedding) showed that HCC patients could be isolated from HBV-infected patients and healthy individuals by comparing the cell-free 5hmC pattern [43]. HCC-specific differentially expressed genes (*q* < 0.001, fold change > 1.41, 1006 genes) could isolate HCC from healthy populations and most HBV samples [44].

**Fig. 3:**
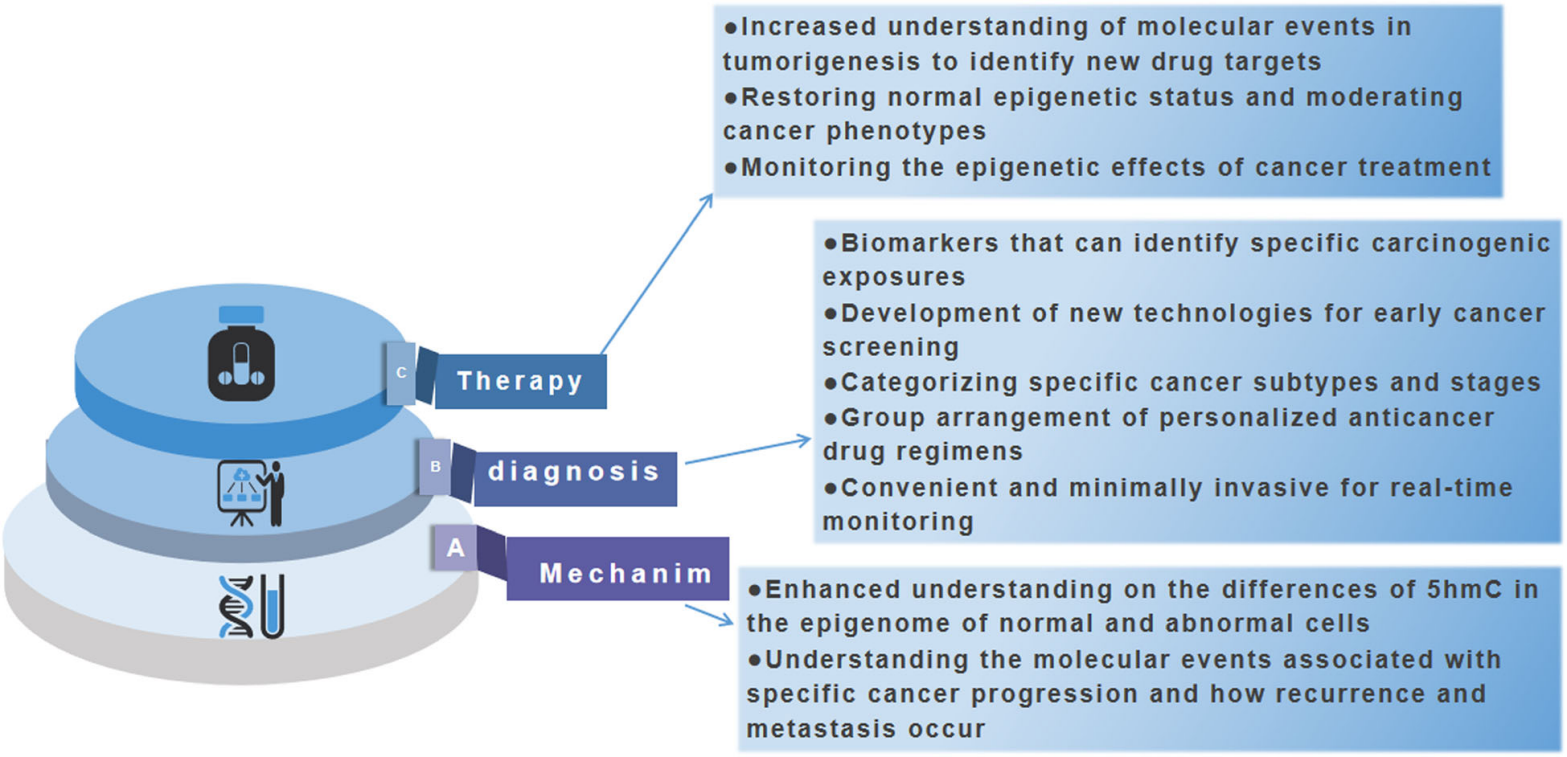
Advances in genome-wide 5-hydroxymethylcytosine spectrum in tumor studies. Genome-wide 5-hydroxymethylcytosine spectrum could advance the field of cancer research. 5hmc-based analysis can help understand the underlying mechanisms associated with cancer progression, identify new diagnostic tools, and provide more effective drug regimens and efficacy monitoring for cancer treatment

Researchers have reported that an improved hMe-seal method makes the quantitative analysis of low levels of 5hmC in cfDNA possible, which has significant advantages over traditional methods [45, 46]. Firstly, hMe-seal does not further degrade cfDNA in samples, unlike the bisulfite method used for cell-free 5mC sequencing. Secondly, compared to genome-wide sequencing methods including mutation sequencing, the concentration of 5hmC not only improves the cost-effectiveness (10–20 million reads,~ 0.5-fold human genome coverage) but more importantly enables the quantitative determination of 5hmC cfDNA from low-frequency tissue sources, such as blood cell samples [33, 46]. In addition to data of the gene body, 5hmC in non-coding regions can also be used as potential biomarkers to predict cancer types [47]. What is noteworthy is that different types of cancer exhibit specific patterns of cell-free hydromethylome, which leads us to think that we can use specific cell-free 5hmC characteristics to predict cancer types with high precision. In the process of step by step clinical application, this will comprehensively analyze and summarize the genetic and epigenetic changes of various tumor states and further enhance the level of individualized diagnosis and precision medicine (Fig 3).

## The potential value of 5hmC in different types of cancer

The traditional view is that a tumor is due to gene mutation and amplification caused by carcinogenic factors, which could lead to the disorder of cell proliferation and differentiation [48]. However, with further understanding of cancer in recent years, researchers found that regulatory mechanisms of the non-coding region perform more critical roles in the occurrence and progression of the tumor [49–51]. For example, DNA methylation, histone modification, and chromatin structure mutation are all found to alter in multiple types of tumors [52, 53]. Researchers found 5mC correlated with the initiation, progression, histological grade, and poor prognosis of human cancers. However, the biological significance of 5hmC in human cancer remains elusive [54]. Discovery of the mechanism for ten-eleven translocation enzymes (TET1, TET2, and TET3), which are capable of the oxidation 5mC to 5hmC and gene regulation, shows that cytosine methylation is essential in mammalian genomic DNA and transcriptional regulation [55, 56]. Global loss of 5hmC, associated with TET downregulation and alteration of TET functions, points to a link between cancer epigenetics and immunoregulation [57, 58].

If the usual way of DNA methylation which is mediated through the coordinated actions of several DNA methyltransferases (DNMTs) that transfer a methyl group from S-adenosyl methionine (SAM) to the carbon-5 position of cytosine does not occur, DNA becomes progressively demethylated through a passive replication-dependent mechanism [59, 60]. This dysregulation happens in both hematological and solid tumors, for example, colon, liver, lung, stomach, skin (melanoma), esophageal squamous cell carcinoma, prostate, blood, and breast tumors [61–63]. The most important thing is that the reduction of TET1 expression appears to be a tumor suppressor gene that can promote the growth and metastasis of cancer [64–66]. Thus, investigating the underlying molecular mechanisms between DNMTs and cancer is of great importance for therapeutic strategies.

### 5hmC and malignant melanoma

Melanoma is a common and aggressive form of cancer, which makes the diagnosis very difficult. Even in the same pathology, the outcomes can vary significantly for lesions [67, 68]. For now, there is only one panel of 31 RNA-based prognostic biomarkers known as Decision Dx-Melanoma that claims to improve prognostic predictions, which is only offered by a single laboratory in the USA [69, 70]. The decreased expression of 5hmC in malignant tissue has been shown consistently in a wide range of different cancers, including melanoma [71–73]. Also, the loss of 5hmC in melanoma has been reported to lead to reduced survival, the decrease of 5hmC was positively correlated with the prognosis of malignant melanoma [74]. At the same time, TET2 reduced the expression in different pathological stages of malignant melanoma [75]. Along with the TET2 reducing, the tumor became more malignant [76, 77].

Clinically, the detection of 5hmC in the skin section of malignant melanoma guides the early diagnosis and prognosis [78]. Besides, 5hmC showed a similarly low level, whether it was from an exposed part [79]. It suggests that the low content of 5hmC may not be correlated with ultraviolet light [80]. These all suggest that 5hmC may be a viable prognostic biomarker in melanoma [81, 82].

### 5hmC and breast cancer

Breast cancer (BC) is a very heterogeneous disease characterized by different molecular and histopathological features; responses to treatment of its subtypes differ based on estrogen receptor, progesterone receptor, and human epidermal growth factor receptor 2 (HER2) status [83, 84]. The complexity has been a big challenge for researchers and clinicians to stratify breast tumors. By evaluating a total of 15 pairs of normal and carcinoma samples in human breast tissue, the results show the levels of 5hmC were dramatically reduced in cancer group compared with the healthy breast tissues [85]. 5hmC of stem cells in breast cancer patients has reduced [86]. The expression of 5hmC was observed by immunohistochemical staining [87]. Researchers found that the nucleus of the unmutated breast cancer cells was 5hmC-positive, while the 5hmC in TET2-mutated tissue was negative [88]. In order to determine the prognostic role of TET family proteins and DNA glycosylase (TDG) in patients with early breast cancer, the expression of mRNAs encoding TET1-3 and TDG in 162 breast cancer tissues was quantified. The result that TET1 mRNA was significantly related to overall survival [89], as well as TET3 and TDG mRNAs were independent prognostic factors for patients, suggests the relationship between DNA methylation and central signaling pathways involved in cancer [90]. There was more lymphocyte infiltration near the tumor cells. The expression of 5hmC in breast cancer of mutated TET2 was decreased [91, 92]. That is to say, abnormal regulation of TET2 may lead the hydroxymethylation of DNA in the wrong way. Furthermore, these all have contributed to finding the epigenetic medication, which can enhance the antitumor immune response.

### 5hmC and bladder cancer

Despite significant advances in surgical techniques and adjuvant therapies, bladder cancer remains a highly prevalent and lethal malignancy [93]. Previous categories of tumor markers include proteomic markers [94, 95], genomic markers, and epigenetic markers [96, 97], and epigenetics is a field that combines genomics and proteomics [98]. The DNA methylation is the most common epigenetic change investigated in bladder tumor markers [99]. 5hmC level has seemed like an independent marker of bladder cancer gradually. In a study of 57 bladder cancer patients and 20 control individuals, by methylation-specific PCR, promoter methylation of E-cadherin, p16, p14, and RASSF1A in DNA isolated from exfoliated cells was evaluated [100].

Consistent with recent findings in different types of cancers, healthy bladder tissues showed intense nuclear 5hmC staining. However, a partial or complete loss of 5hmC was shown in bladder cancer. Patients with higher 5hmC levels had lived longer than the ones with lower levels; meanwhile, the lower 5hmC level, the higher stage and metastasis of the tumor. 5hmC is found to appear in gene-rich regions of the bladder in the normal genome and is correspondingly low in the cancer genome. The loss of 5hmC occurred in different cancer-related genes during bladder carcinogenesis. As the expression of TET2, L2HGDH, and vitamin C transporters (SVCT1 and SVCT2) was decreased in all bladder cancer cell lines, vitamin C is found to be capable of increasing 5hmC as a cofactor of TET proteins [101]. The researchers then treated several bladder cancer cell lines with vitamin C at various concentrations and periods. It turned out that vitamin C increased the 5hmC levels in T24 cells, which depends on time and concentration. Moreover, similar effects were observed that 5hmC levels increased with vitamin C, which inhibited cell growth in renal cancer [102]. It is also indicated that vitamin C can increase 5hmC levels in bladder cancer cells. Importantly, researchers observed that 5hmC promotes the activity of TET enzymes rather than increase the expression levels directly. It was also reported that high-dose vitamin C could restrain cancer cells by producing H_2_O_2_ [4], unlike low-dose vitamin C, which suppressed cancer growth in an H_2_O_2_-independent way that included 5hmC restoration.

At present, there has been much research on 5hmC in the tumor. However, their specific function and mechanism in various cancers are not clear. Therefore, analyzing cancer-related genome-wide hydroxymethylated regions and binding sites of TET proteins will help to figure out their importance in cancer.

## Discussion

DNA methylation is a process in which cytosine (C) is catalyzed by DNA methyltransferase to produce 5mC, and the product is called the “fifth base” of DNA. DNA hydromethylation refers to the oxidation of 5mC by TET to produce 5hmC, the “sixth base” of DNA [103]. It is believed that methylation can inactive some genes, leading to gene silencing. However, demethylation can cause gene reactivation and expression, and the content of DNA methylation in tumors of different systems is still controversial. Studies have shown that the distribution of 5hmC is tissue-specific, and there are differences in the distribution of 5hmC in different organs and tissues. Also, the content of 5hmC in tissues and organs is affected by complex factors such as environmental toxins and the internal environment of the human body.

Because of the convenience of obtaining cfDNA and the smaller trauma to the human body, the study on 5hmC in cfDNA has attracted extensive attention from researchers. The acquisition and analysis of 5hmC in cfDNA provide a new perspective for the early diagnosis, effective treatment, and prognosis evaluation of tumors. With the development of biological information technology and high-throughput sequencing technology, the quantitative techniques of hydroxymethylation analysis such as hMeDIP-seq and TAB-Seq are becoming more and more mature, providing an advantageous method for DNA hydroxymethylation modification research in the tumor. The exploration of tumor-related genome-wide methylation regions is conducive to the in-depth understanding of the role of DNA hydromethylation modification in the development of solid tumors and hematological tumors, to find new tumor molecular markers and therapeutic targets, which may become a research hotspot in the next stage of methylation modified tumors.

In recent years, researches on hydromethylation mainly focus on tumors and psychiatric diseases [104, 105]. Studies have shown that during organ maturation and drug-induced response, 5hmC analysis can be used as an indicator of cell status and contribute to the identification of toxic carcinogen exposure [106, 107]. Dynamic enrichment patterns of methylation and hydromethylation markers may enable us to identify target genes for epigenetic modifications associated with prostate cancer [108]. Through a comprehensive analysis of global DNA methylation and hydroxymethylation in tumors and healthy tissues, colorectal cancer-related genes can be screened [109]. By changing the DNA methylation state, 5hmC can prevent the inactivation of some tumor suppressor genes and apoptotic genes. The decreased level of 5hmC leads to the lack of gene protection and the occurrence of tumors.

Although studies have suggested that methylation modification is associated with tumorigenesis and progression, studies on hydromethylation in ctDNA are still lacking. Moreover, the mechanism by which DNA methylation and hydroxymethylation affect oncogenesis and progression in different systems has not been clearly studied, and the specific role of epigenetic modification needs to be further explored. However, due to the complexity of biopsy required for the diagnosis of most tumors and cfDNA content in peripheral blood samples is susceptible to many external factors, it remains to be further studied whether other methods can be used to replace puncture biopsy in the future. What is the preference of hydromethylation in tumor-related genes for CpG sites? Which TET enzyme is involved in hydroxymethylation modification? How does hydromethylation status of specific gene promoters change after treatment? These problems need to be further explored.

## Conclusions

As the intermediate product of cytosine modification, 5hmC has offered a broad perspective of the epigenetic regulation process. 5hmC is a prospective marker, which correlates to different stages of cancer. Future research may be inclined to explain the connection between 5hmC and intractable diseases, especially in early detection of tumors and the prediction of chemotherapeutic resistance. As far as the environment is concerned, its variety influences epigenetic modification, so as environmental toxins. After understanding the changes in 5hmC, we will get more details about abnormal reactions caused by DNA hydroxymethylation. The relationship between 5hmC and the human body is expected to be a current issue in future research.

## Data Availability

The datasets generated/analyzed during the current study are available.
